# Impact on mortality and cancer incidence rates of using random invitation from population registers for recruitment to trials

**DOI:** 10.1186/1745-6215-12-61

**Published:** 2011-03-01

**Authors:** Matthew Burnell, Aleksandra Gentry-Maharaj, Andy Ryan, Sophia Apostolidou, Mariam Habib, Jatinderpal Kalsi, Steven Skates, Mahesh Parmar, Mourad W Seif, Nazar N Amso, Keith Godfrey, David Oram, Jonathan Herod, Karin Williamson, Howard Jenkins, Tim Mould, Robert Woolas, John Murdoch, Stephen Dobbs, Simon Leeson, Derek Cruickshank, Stuart Campbell, Lesley Fallowfield, Ian Jacobs, Usha Menon

**Affiliations:** 1 Gynaecological Oncology, UCL EGA Institute for Women's Health, London W1T 7DN, UK; 2 Department of Medicine, Harvard Medical School, Boston, MA, US MA 02115, USA; 3 Medical Research Council Clinical Trials Unit, London NW1 2DA, UK; 4 Academic Unit of Obstetrics and Gynaecology, St. Mary's Hospital, Manchester M13 9WL, UK; 5 Department of Obstetrics and Gynaecology, Wales College of Medicine, Cardiff University, Cardiff CF14 4XN, UK; 6 Northern Gynaecological Oncology Centre, Queen Elizabeth Hospital, Gateshead NE9 6SX, UK; 7 Department of Gynaecological Oncology, St. Bartholomew's Hospital, London EC1A 7BE, UK; 8 Department of Gynaecology, Liverpool Women's Hospital, Liverpool L8 7SS, UK; 9 Department of Gynaecological Oncology, Nottingham City Hospital, Nottingham NG5 1PB, UK; 10 Department of Gynaecological Oncology, Derby City Hospital, Derby DE22 3NE, UK; 11 Department of Gynaecological Oncology, Royal Free Hospital, London NW3 2QG, UK; 12 Department of Gynaecological Oncology, St. Mary's Hospital, Portsmouth PO3 6AD, UK; 13 Department of Gynaecological Oncology, St. Michael's Hospital, Bristol BS2 8EG, UK; 14 Department of Gynaecological Oncology, Belfast City Hospital, Belfast BT9 7AB, UK; 15 Department of Gynaecological Oncology, Llandudno Hospital, North Wales LL30 1LB, UK; 16 Department of Gynaecological Oncology, James Cook University Hospital, Middlesbrough TS4 3BW, UK; 17 Create Health Clinic, London W1G 6AJ, UK; 18 Psychosocial Oncology Group, University of Sussex, Brighton BN1 9QG, UK

## Abstract

**Background:**

Participants in trials evaluating preventive interventions such as screening are on average healthier than the general population. To decrease this 'healthy volunteer effect' (HVE) women were randomly invited from population registers to participate in the United Kingdom Collaborative Trial of Ovarian Cancer Screening (UKCTOCS) and not allowed to self refer. This report assesses the extent of the HVE still prevalent in UKCTOCS and considers how certain shortfalls in mortality and incidence can be related to differences in socioeconomic status.

**Methods:**

Between 2001 and 2005, 202 638 postmenopausal women joined the trial out of 1 243 312 women randomly invited from local health authority registers. The cohort was flagged for deaths and cancer registrations and mean follow up at censoring was 5.55 years for mortality, and 2.58 years for cancer incidence. Overall and cause-specific Standardised Mortality Ratios (SMRs) and Standardised Incidence Ratios (SIRs) were calculated based on national mortality (2005) and cancer incidence (2006) statistics. The Index of Multiple Deprivation (IMD 2007) was used to assess the link between socioeconomic status and mortality/cancer incidence, and differences between the invited and recruited populations.

**Results:**

The SMR for all trial participants was 37%. By subgroup, the SMRs were higher for: younger age groups, extremes of BMI distribution and with each increasing year in trial. There was a clear trend between lower socioeconomic status and increased mortality but less pronounced with incidence. While the invited population had higher mean IMD scores (more deprived) than the national average, those who joined the trial were less deprived.

**Conclusions:**

Recruitment to screening trials through invitation from population registers does not prevent a pronounced HVE on mortality. The impact on cancer incidence is much smaller. Similar shortfalls can be expected in other screening RCTs and it maybe prudent to use the various mortality and incidence rates presented as guides for calculating event rates and power in RCTs involving women.

**Trial Registration:**

This study is registered as an International Standard Randomised Controlled Trial, number ISRCTN22488978.

Medical Research Council (grant no. G990102), Cancer Research UK (grant no. C1479/A2884) and Department of Health

## Background

In clinical studies, mortality and morbidity data from the general population is used to calculate expected death and incidence rates. However, volunteers participating in trials evaluating preventive interventions such as screening are on average healthier than the general population [1-3]. The implication of this 'healthy volunteer effect (HVE)' is that trial participants have lower mortality and morbidity than the general population. In randomised controlled trials (RCTs), this can cause a shortfall in expected event rates which are the foundation of the trial's power calculations [4]. The latter determine the sample size (number of participants recruited and their time on the trial) and contribute significantly to design, logistics and cost. A deficit could mean a significant fall in power and may require alteration of the design midway through the trial if the primary objective is to be achieved.

The United Kingdom Collaborative Trial of Ovarian Cancer Screening (UKCTOCS) is a large multi-centre randomised controlled trial of 202 638 women recruited between 2001 and 2005 [5]. In order to ensure trial volunteers were as representative of the general population as possible, women were not allowed to self refer. Instead over 1.2 million women aged 50-74 were randomly invited from age sex registers of 27 participating local health authority registers [5]. The underlying hypothesis was that the HVE is largely related to socioeconomic status with participants being more affluent, better educated and more health-conscious than the population as a whole. This bias was thought to be magnified by recruitment using self-referral, which is dependent on publicising the trial through a variety of media such as newspapers, magazines, radio, television, posters at numerous venues and meetings.

In this paper, we report on the impact of population invitation on the HVE in UKCTOCS by comparing observed and expected mortality and cancer incidence rates in the trial, particularly with regard to socioeconomic status levels. We also consider the differences in deprivation of those invited with those recruited. The results provide vital data to inform trial design and sample size calculations for those seeking to undertake screening studies involving the general population.

## Methods

### Study Design

UKCTOCS is an RCT aiming to assess the impact of screening on ovarian cancer mortality while comprehensively evaluating performance characteristics, physical and psychological morbidity, compliance, and cost of the screening strategies. It was set up in 13 NHS Trusts in England, Wales and Northern Ireland. Women living in adjoining Primary Care Trusts (including Local Health Boards in Wales) were invited to participate in the trial. Those who accepted the invitation attended a local recruitment clinic. Detailed description of the invitation and recruitment process, as well as inclusion/exclusion criteria, are detailed elsewhere [5]. Of relevance to this analysis is that women with an active malignancy were only eligible if they had no documented persistent or recurrent disease, and those with previous history of ovarian cancer were excluded. All women provided written consent.

### Follow up

Women recruited into the trial were 'flagged' for follow up with the NHS Information Centre for Health and Social Care (ICHSC) in England and Wales (for death and cancer registration) and with the Central Services Agency (CSA, for deaths in Northern Ireland) and Cancer Registry in Northern Ireland (NICR). Almost all women were successfully flagged (*n *= 202 593). From the received death certificate copies, the 'underlying cause of death' was used for the cause-specific observed counts. Barring an inquest, death certificates were mostly received within 3 months of the death. To ensure completeness of data on deaths, events were censored on the 1^st ^June 2009, eight months prior to the last death certificates update on 1^st ^February 2010. Data provided by the CSA on cause of death was incomplete and therefore women from Northern Ireland were excluded from the calculation of cause-specific SMRs.

Information on all incident cancers can take up to 3 years to be recorded with the national registries. In order to ensure completeness of data on cancers, events were censored on 1^st ^June 2006, allowing a time lag of 3.75 years between events and the final cancer registration update from NHS ICHSC and the NICR in February 2010. Unlike the CSA, the NICR provided full data on cancer type, so that all women were included in the cancer-specific incidence analysis.

### Analysis

#### Mortality and cancer incidence

Evidence of a HVE was assessed by calculation of the Standardised Mortality Ratio (SMR) which is defined as the ratio of observed to expected deaths (×100). A value significantly less than 100 would indicate a HVE. SMRs were calculated for: overall mortality (including ovarian cancer); overall cancer (excluding ovarian cancer and tubal cancer (ICD10-C56, C57.0) and 'other malignant neoplasms of skin' (ICD10-C44); the 10 leading individual causes of female cancer mortality (excluding ovarian cancer); and the five leading general causes other than cancer (circulatory, respiratory, digestive, nervous system and mental and behavioural). Except for overall mortality, ovarian and fallopian tube cancers were excluded from the analysis as they were the primary outcome measure of the ongoing RCT.

The effect on cancer incidence from the HVE was similarly assessed with the Standardised Incidence Ratio (SIR), defined as the ratio of observed to expected cancer incidence (×100). This was calculated again for overall cancer (also excluding ICD10-C56, C57.0 and C44) and the leading 10 female causes of cancer incidence, excluding ovarian cancer. These are the same as for mortality, although there are differences in the rankings.

For each trial participant, Expected Mortality Rates (EMRs) were calculated using national mortality rates derived from ONS for 2005 [6]. All individual EMRs were calculated for each year or partial year on the trial, with the values summed over the number of years on the trial. The individual's risk of mortality was adjusted for age at randomisation and also dynamically, so that the risk reflected the ageing woman as the screening progressed. The overall EMR for a cause was the sum of each woman's individual EMR up to the censoring date of 1^st ^June 2009. ONS mortality tables for 2005 [6] provide both the number of female deaths for each cause as well as total female population in 5 year age groups. To estimate an age-specific mortality rate for each year, firstly the midpoint of the age group was taken as representing the mortality rate calculated for that age group. An approximate mortality rate estimate for any given age was then calculated by imputing the age into either a best fitting quadratic or exponential function. For nearly all causes the fit was excellent with *R*^2 ^always over 0.95 and mostly over 0.99. Similar analysis was performed for cancer incidence using ONS cancer incidence tables for 2006 [7]. The only exception was breast cancer incidence where the effect of a national screening programme meant that after the age of 70 the incidence fell sharply, so that 2 separate functions had to be used (below and above age 70). All confidence intervals for the SMRs and SIRs were based on an assumed poisson distribution for the observed deaths or cancers.

To calculate the EMR of each woman *i *(*i *= 1, 2...202 593) for cause of death *z *if:

• *t *is the year on trial (*t *= 1, 2...8)

• *x_i _*is the age of woman *i *at randomisation

• *D_zx _*is the imputed mortality rate for cause of death *z *at age *x*

• *y_ti _*is the fraction of the year of trial *t *completed at censoring or death by woman *i *(always = 1, except for most recent/last year on trial)

then

$$EMR_{iz}=\sum_{t=1,2...8}[D_{z(x_{i}+t-0.5)}]y_{ti}$$

and the overall EMR for cause of death *z *is simply:

$$EMR_{z}=\sum_{i=1,2...202593}EMR_{iz}$$

Note that the age *x *imputed in *D_zx _*is slightly adjusted by 0.5 to approximate the average effect of ageing over the year (*t *- 0.5 instead of *t*-1). Also note, if women withdraw from attending for screening in the trial at any point, they continue to be followed up through flagging for death and cancer registration. Hence no adjustment is made for withdrawals.

#### Socioeconomic status

The PCT provided postcodes and dates of birth for all women invited to the trial. The former was used to estimate socio-economic class. The Index of Multiple Deprivation 2007 (IMD) [8] provides 32 482 scores at a Super Output Area (SOA) level linked to postcodes for England. It was chosen over the other two census based available indices (Townsend or Carstairs) as firstly, the most up-to-date and secondly, the most precise in ascribing a score to an individual based on postcode, given that it is calculated at a much finer spatial scale. Upon linking, an individual IMD score was derived for 156 620 women recruited in England. The women recruited from centres in Wales and Northern Ireland were omitted from this particular analysis. A Welsh IMD (2008) [9] has been published. However, the differing criteria employed make comparisons between the English and Welsh IMD scores invalid [10]. To explore mortality versus deprivation, the recruited women were separated into quintiles according to IMD score and the respective SMRs compared. This was also repeated for cancer incidence.

IMD scores for all women who were invited from England were compared with those recruited to the trial by evaluating their relative frequency distributions. No mortality data is available for invited women.

#### Variation in HVE with age/region/BMI/time on trial

For all these analyses, the expected and observed mortality rates for all relevant women in each group were summed. Regional variations were compared by summing over the individual recruitment centres. For the age group analysis the groupings were made by using the age at randomisation to categorise into 50-54, 55-59, 60-65, 65-69 or 70-74 age groups. To assess any change in the SMR/SIRs over the trial period, the overall EMR was partitioned into year in trial by summing the individual EMRs for each year *t*. This was done for the individual causes of mortality and incidence, as well as for overall mortality. Mortality versus body mass index (BMI) of the women was explored by separating women who provided height and weight at randomisation into the standard underweight, normal, overweight and obese categories (up to 18.5; 18.5-25; 25-30; over 30, respectively) and comparing respective SMRs.

## Results

1 243 282 women residing in 27 Primary Care Trusts (including Local Health Boards in Wales) adjoining the 13 trial centres in England (10), Wales (2) and Northern Ireland (1) were invited to participate in the trial. Between 17th April 2001 and 29th September 2005, 202 638 women (157 973 England, 31 086 Wales, 13 579 Northern Ireland) were recruited and randomised [5]. Of those recruited from England and Wales, 35 were unsuccessfully matched and 10 refused consent for flagging, leaving 189 014 women from England and Wales undergoing flagging through NHS ICHSC. All 13 579 women recruited from Northern Ireland were successfully matched by the Northern Ireland CSA. The average number of years on trial when mortality events were censored on 1^st ^June 2009 for mortality was 5.55 years, with over 99% having been on the trial for over 3 years, and 24% over 7 years. Mean follow-up for incidence was 2.58 years at 1^st ^June 2006.

### Mortality rates

There were only 4554 observed deaths compared to the expected number of 12 247 based on 2005 national mortality rates (Table 1). The SMR for overall mortality was 37.3% (95% CI: 36.2, 38.4%). There was some variation of SMR across the 13 trial centres with the highest, 48.4% at Liverpool and the lowest, 30.9% at Bristol. However across all centres there was a strong HVE with less than half the expected deaths (Table 1).

**Table 1:** Standardized Mortality Ratios (SMR) for overall mortality and by subgroup.

	Number	Expected Mortality	Observed Mortality	SMR	*L95%CI*	*U95%CI*
**OVERALL**	**202593**	**12247**	**4569**	**37.3%**	***36.2%***	***38.4%***
CENTRE						
Gateshead	17321	1140	469	41.1%	*37.5%*	*45.0%*
St Barts London	19963	1199	474	39.5%	*36.0%*	*43.2%*
Liverpool	10108	613	297	48.4%	*43.1%*	*54.3%*
Nottingham	16765	1091	430	39.4%	*35.8%*	*43.3%*
Manchester	16498	940	375	39.9%	*35.9%*	*44.1%*
Derby	14917	858	312	36.4%	*32.5%*	*40.7%*
Royal Free London	16709	920	306	33.3%	*29.7%*	*37.2%*
Portsmouth	19175	1341	490	36.5%	*33.4%*	*39.9%*
Bristol	16556	1108	342	30.9%	*27.7%*	*34.3%*
Belfast	13579	750	241	32.2%	*28.2%*	*36.5%*
Cardiff	16756	886	338	38.2%	*34.2%*	*42.5%*
North Wales	14324	861	298	34.6%	*30.8%*	*38.8%*
Middleborough	9922	541	197	36.4%	*31.5%*	*41.9%*
AGE GROUP						
50-54	39392	882	417	47.3%	*42.8%*	*52.0%*
55-59	55592	1977	825	41.7%	*38.9%*	*44.7%*
60-64	47073	2658	948	35.7%	*33.4%*	*38.0%*
65-69	38568	3530	1259	35.7%	*33.7%*	*37.7%*
70-74	21967	3200	1120	35.0%	*33.0%*	*37.1%*
BMI SCORE						
Underweight (up to 18.5)	2548	157	111	70.6%	*58.1%*	*85.0%*
Normal (18.5-25)	82888	4903	1701	34.7%	*33.1%*	*36.4%*
Overweight (25-30)	74284	4640	1586	34.2%	*32.5%*	*35.9%*
Obese (over 30)	41903	2486	1113	44.8%	*42.2%*	*47.5%*
YEAR IN TRIAL	(women years)					
1^st^	202501	1732	320	18.5%	*16.5%*	*20.6%*
2^nd^	202028	1914	660	34.5%	*31.9%*	*37.2%*
3^rd^	201314	2111	800	37.9%	*35.3%*	*40.6%*
4^th^	197029	2290	903	39.4%	*36.9%*	*42.1%*
5^th^	156319	1955	841	43.0%	*40.2%*	*46.0%*
6^th^	106119	1393	631	45.3%	*41.8%*	*49.0%*
7^th^	49590	719	349	48.6%	*43.6%*	*53.9%*
8^th^	8136	133	65	49.0%	*37.9%*	*62.5%*

Average time on trial = 5.55 yrs/woman

For age group, there was an apparent decrease in the SMRs as age increased, with the youngest group (50-54; SMR = 47.3%) having a less pronounced HVE than the other groups (Table 1). For BMI, both normal and overweight categories had similar SMRs of around 34% whereas the extreme categories had higher mortality rates, particularly the underweight category (70.6%)

The overall cancer SMR was 55.9%. There was some variation between the different cancer types but all were between 42.9% (breast) and 79.8% (pancreatic), with mortality significantly lower than expected (100%). The HVE was even stronger for the other major causes of mortality (Table 2).

**Table 2:** Standardized Mortality Ratios (SMRs) for overall cancer, the 10 leading causes of cancer mortality and 5 other causes of mortality.

MORTALITY CAUSE	ICD-10 Code	Expected mortality	Observed Mortality	SMR	*L95%CI*	*U95%CI*
Cancers Overall	C00:C99 excl. C56, C57.0, C44	4419	2469	55.9%	*53.7%*	*58.1%*
Lung Cancer	C33:C34	1020	499	48.9%	*44.7%*	*53.4%*
Breast Cancer	C50	813	349	42.9%	*38.6%*	*47.7%*
Colorectal Cancer	C18:C21	415	218	52.6%	*45.8%*	*60.1%*
Pancreatic Cancer	C25	244	195	79.8%	*69.0%*	*91.8%*
Oesophagus Cancer	C15	111	85	76.4%	*61.0%*	*94.5%*
Stomach Cancer	C16	85	64	75.1%	*57.8%*	*95.9%*
N-H Lymphoma	C82:C85, C96	194	88	45.4%	*36.4%*	*55.9%*
Leukaemia	C91:C95	107	49	45.9%	*33.9%*	*60.6%*
Uterus Cancer	C54:C55	120	63	52.3%	*40.2%*	*66.9%*
Bladder Cancer	C67	68	32	46.7%	*32.0%*	*66.0%*
						
Mental Behaviours Deaths	F-	127	9	7.1%	*3.2%*	*13.4%*
Nervous System Deaths	G-	344	92	26.8%	*21.6%*	*32.8%*
Circulatory Deaths	I-	3208	999	31.1%	*29.2%*	*33.1%*
Respiratory Deaths	J-	1179	261	22.1%	*19.5%*	*25.0%*
Digestive Deaths	K-	688	187	27.2%	*23.4%*	*31.4%*

Ovarian (C56)/tubal cancer (C57.0) and other malignant neoplasm of skin (C44) have been excluded. Average time on trial at censoring (1^st ^June 2009) = 5.55 yrs/woman

There was a clear increase in the SMRs as time in trial increased (Table 3). The overall SMR was low in the 1^st ^year (18.5%) but rose steadily to 49.0% by the 8^th ^year. With the exception of stomach cancer all of the cause-specific 1^st ^year SMRs for mortality were significantly below 100% with some particularly low values such as 5.3% for breast cancer (Table 3). These figures showed an increasing trend as the study progressed, though not nearly as consistently as for overall mortality, and given the lower numbers, with wider confidence intervals. Of the cancers only lung, breast and colorectal had 6 or more study years where the confidence interval for the SMR did not contain 100.

**Table 3:** Standardized Mortality Ratios for various causes by year in trial.

Year of Study	Year 1	Year 2	Year 3	Year 4	Year 5	Year 6	Year 7	Year 8	Overall
*Women years*	*188926*	*188467*	*187776*	*183826*	*146094*	*99763*	*46820*	*8057*	***1049731***
**Overall**	**18.5%**	**34.5%**	**37.9%**	**39.4%**	**43.0%**	**45.3%**	**48.6%**	**49.0%**	***37.3%***
Cancers Overall	*19.7%*	*49.7%*	*61.3%*	*59.9%*	*70.4%*	*74.7%*	*64.8%*	*55.8%*	***55.9%***
Lung Cancer	*23.7%*	*37.7%*	*62.4%*	*51.9%*	*63.9%*	*48.3%*	*61.9%*	*18.9%*	***48.9%***
Breast Cancer	*5.3%*	*39.2%*	*37.6%*	*48.4%*	*67.4%*	*72.8%*	*43.8%*	40.4%	***42.9%***
Colorectal Cancer	*18.4%*	*57.9%*	*59.9%*	*54.4%*	*59.8%*	75.5%	*33.6%*	44.1%	***52.6%***
Pancreatic Cancer	27.5%	78.5%	98.3%	*57.4%*	89.6%	138.2%	96.6%	83.4%	***79.8%***
Oesophagus Cancer	14.7%	64.0%	84.2%	91.1%	85.2%	151.7%	108.2%	124.1%	***76.4%***
Stomach Cancer	62.9%	117.8%	68.9%	73.9%	85.4%	*19.8%*	93.3%	94.6%	***75.1%***
N-H Lymphoma	*10.5%*	*35.3%*	*41.4%*	*44.6%*	*53.2%*	70.4%	111.3%	49.5%	***45.4%***
Leukaemia	*6.4%*	*46.8%*	*43.0%*	*30.2%*	*48.0%*	109.8%	49.9%	176.6%	***45.9%***
Uterus Cancer	*11.0%*	66.2%	*42.6%*	67.5%	75.7%	68.8%	*0.0%*	83.1%	***52.3%***
Bladder Cancer	*10.9%*	*19.3%*	60.1%	61.7%	53.6%	49.8%	70.5%	118.6%	***46.7%***
									
Mental Behaviours Deaths	*6.7%*	*0.0%*	*4.8%*	*4.1%*	*4.5%*	*6.2%*	*22.4%*	107.0%	***7.1%***
Nervous System Deaths	*6.1%*	*22.2%*	*20.2%*	*31.2%*	*27.5%*	*43.8%*	59.9%	25.9%	***26.8%***
Circulatory Deaths	*25.7%*	*31.7%*	*31.0%*	*31.9%*	*32.8%*	*26.7%*	*41.2%*	*38.6%*	***31.1%***
Respiratory Deaths	*9.1%*	*16.6%*	*18.6%*	*23.7%*	*25.1%*	*29.7%*	*35.7%*	65.9%	***22.1%***
Digestive Deaths	*15.7%*	*29.9%*	*22.6%*	*27.5%*	*24.3%*	*36.9%*	*41.5%*	82.2%	***27.2%***

Ovarian (C56)/tubal cancer (C57.0) and other malignant neoplasm of skin (C44) have been excluded. Average time on trial at censoring (1^st ^June 2009) = 5.55 yrs/woman. Note the number of 'Women years' relates to individual causes and not to overall mortality due to exclusion of Northern Ireland women. Italicised values have 95% confidence intervals not containing 100.

### Socioeconomic status comparison

**Figure 1:**
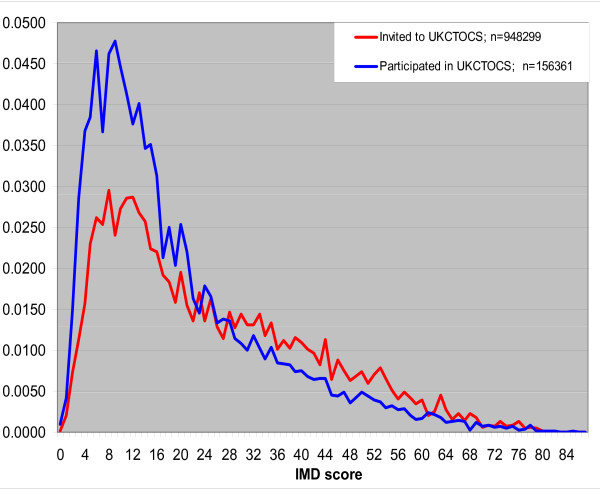
**Relative frequency distributions of IMD score for those invited to UKCTOCS and those that joined UKCTOCS (English postcodes only)**.

Based on IMD score quintiles, there was a general trend between deprivation and mortality with higher SMRs for increasing levels of deprivation (Table 4). Specifically, the lowest (least deprived) two quintiles had a similar SMR of around 30% with the most deprived having a SMR of 52.3%. The rising trend between overall cancer incidence and increasing deprivation was less obvious. Figure 1 shows the relative frequency distributions for IMD score. Although the distributions show similarity in trend and location, the more peaked distribution of those who joined, and the crossover of distributions at an IMD score of 20, imply that the trial participants were less deprived than the invited population.

**Table 4:** Standardized Mortality Ratios (SMR) and Standardized Incidence Ratios (SIR) by deprivation (IMD) quintile.

Mortality by deprivation	Number	Expected Mortality	Observed Mortality	SMR	*L95%CI*	*U95%CI*
IMD SCORE (deprivation index)						
1st quintile (up to 7.8)	31231	1985	604	30.4%	*28.0%*	*33.0%*
2nd quintile (7.8-12.3)	31203	1912	591	30.9%	*28.5%*	*33.5%*
3rd quintile (12.3-18.5)	31254	1884	721	38.3%	*35.5%*	*41.2%*
4th quintile (18.5-30.2)	31326	1901	707	37.2%	*34.5%*	*40.0%*
5th quintile (over 30.2)	31263	1959	1024	52.3%	*49.1%*	*55.6%*
						

**Cancer incidence by deprivation**	**Number**	**Expected Incidence**	**Observed Incidence**	**SIR**	***L95%CI***	***U95%CI***

IMD SCORE (deprivation index)						
1st quintile (up to 7.8)	31231	736	593	80.6%	*74.2%*	*87.4%*
2nd quintile (7.8-12.3)	31203	715	590	82.6%	*76.0%*	*89.5%*
3rd quintile (12.3-18.5)	31254	705	601	85.2%	*78.5%*	*92.3%*
4th quintile (18.5-30.2)	31326	725	589	81.3%	*74.9%*	*88.1%*
5th quintile (over 30.2)	31263	786	715	91.0%	*84.4%*	*97.9%*

Average time on trial = 5.55 yrs/woman for SMRs and = 2.58 yrs/woman for SIRs

### Cancer Incidence

The situation regarding cancer incidence (Table 5) was rather different. For overall cancer the SIR was 88.1%, higher than the 55.9% for overall cancer mortality. Of the individual cancers, only for lung, pancreatic, oesophageal and colorectal cancers did the confidence interval for the SIR not contain 100, and only for pancreatic and oesophageal cancer was the SMR higher than the SIR.

**Table 5:** Standardized Incidence Ratios (SIRs) for overall cancer and the 10 leading causes of cancer.

INCIDENCE	ICD-10 Code	Expected incidence	Observed Incidence	SIR	*L95%CI*	*U95%CI*
Cancers Overall	C00:C99 excl. C56, C57.0, C44	4610	4131	88.1%	*85.4%*	*90.9%*
Lung Cancer	C33:C34	582	328	56.2%	*50.2%*	*62.6%*
Breast Cancer	C50	1715	1748	99.8%	*95.1%*	*104.6%*
Colorectal Cancer	C18:C21	511	471	91.1%	*83.1%*	*99.8%*
Pancreatic Cancer	C25	122	84	68.1%	*54.3%*	*84.4%*
Oesophagus Cancer	C15	75	54	71.6%	*53.8%*	*93.4%*
Stomach Cancer	C16	65	56	85.0%	*64.0%*	*110.6%*
N-H Lymphoma	C82:C85, C96	168	175	103.6%	*88.8%*	*120.2%*
Leukaemia	C91:C95	75	58	77.7%	*59.0%*	*100.4%*
Uterus Cancer	C54:C55	318	293	91.1%	*80.9%*	*102.2%*
Bladder Cancer	C67	77	64	79.5%	*60.8%*	*102.1%*

Ovarian (C56)/tubal cancer (C57.0) and other malignant neoplasm of skin (C44) have been excluded. Average time in trial at censoring (1^st ^June 2006) = 2.58 yrs/woman.

Regarding incidence over time (Table 6), there were far fewer occasions compared to mortality where the whole confidence interval for the SIR was below 100, with only lung cancer having a consistently low SIR over time (between 27.1% and 65.9%). Apart from lung cancer and leukaemia, the cancer specific SIRs were not particularly low in the first year compared to the complete study period. For overall cancer the SIRs were remarkably consistent over time, between 84.6% and 91.7%.

**Table 6:** Standardized Incidence Ratios for 10 leading causes of cancer by year in trial.

Year of Study	Year 1	Year 2	Year 3	Year 4	Year 5	Overall
*Women years*	*198967*	*158235*	*107587*	*50362*	*8296*	***523447***
Cancers Overall (excl. C56, C57.0, C44)	*84.6%*	*90.8%*	*88.5%*	91.7%	91.4%	***88.1%***
Lung Cancer	*45.0%*	*61.5%*	*65.9%*	*64.1%*	*27.1%*	***56.2%***
Breast Cancer	100.0%	106.1%	90.5%	99.9%	95.3%	**99.8%**
Colorectal Cancer	87.2%	96.7%	84.5%	98.4%	112.0%	***91.1%***
Pancreatic Cancer	*57.4%*	*65.4%*	88.4%	83.5%	0.0%	***68.1%***
Oesophagus Cancer	73.8%	61.6%	99.8%	37.1%	68.8%	***71.6%***
Stomach Cancer	78.5%	102.6%	72.2%	70.2%	152.7%	**85.0%**
N-H Lymphoma	91.5%	92.7%	137.8%	102.7%	131.1%	**103.6%**
Leukaemia	*44.6%*	75.5%	101.0%	138.1%	140.0%	**77.7%**
Uterus Cancer	96.3%	92.7%	94.1%	*58.7%*	109.2%	**91.1%**
Bladder Cancer	*59.0%*	90.9%	103.2%	82.6%	0.0%	**79.5%**

Ovarian (C56)/tubal cancer (C57.0) and other malignant neoplasm of skin (C44) have been excluded. Average time in trial at censoring (1^st ^June 2006) = 2.58 yrs/woman. Italicised values have 95% confidence intervals not containing 100.

## Discussion

This is the first report to explore the impact of a 'healthy volunteer effect' from inviting potential participants randomly from population registers as opposed to self-referral. The overall SMR compared to the 2005 population of England and Wales was 37.3%. The figure is almost identical to the overall SMR of 38% reported for women in the US PLCO screening trial [2] where participants were allowed to self refer or invited through mass mailings using motor-vehicle registrations and health care organization lists which were not generally population based [11]. This introduces additional bias, as the types of advertising media (radio station, website, newspaper or magazine) or mailing lists used, limit those who have access to the information. In contrast, in UKCTOCS over 1.2 million women (1 in 6 of the UK population in the eligible age range) were randomly invited from health authority registers. It was anticipated that such invitation would result in participants being more representative of the general population than those recruited through advertisement and self referral. However, even with this safeguard there continued to be a pronounced HVE on mortality, both overall and cause-specific. The data highlights again the selection bias that occurs in clinical trials and emphasises the need for randomised controlled trials rather than observational studies to determine efficacy of screening and prevention strategies. There was a much lesser effect on cancer incidence relative to the general population.

The magnitude of the HVE in a trial is dependent on a variety of other factors in addition to mode of recruitment. Eligibility criteria can play a crucial role. This includes gender, where in the PLCO trial the HVE was less pronounced in men, who had a statistically significantly higher SMR than women for all-cause mortality (46% versus 38%), all cancer mortality, respiratory diseases, diabetes, cardiovascular diseases, and non-Hodgkin's lymphoma [2]. Most screening/prevention trials exclude those with an ongoing active malignancy. This will inevitably affect the cancer specific SMRs in the early years. There was a clear upward trend in the cancer specific SMRs, despite widening confidence intervals, when examined by 'year in trial'. In the first year, most were below 25%. It is feasible that the other exclusion criteria may also have had health implications. Women who had undergone bilateral oophorectomy were ineligible. Recent reports have shown increased mortality in women in this subgroup who do not use oestrogen replacement until the age of 45 [12,13]. Finally, higher participations rates may be expected to reduce the self-selection effect. In UKCTOCS 25% of women invited replied that they would like to participate in the trial but finally only 16% were randomised [5].

The overall 1^st ^year SMR was 18.5%, rising to 35% in the 2^nd ^year and nearly 50% by the 8^th ^year. The SMRs have been age-adjusted dynamically so this is not a result of the age-related increase in risk. Similar trends were seen in the PLCO trial. In both studies by the 7^th ^year the SMR was 48%. A major contributing factor to the SMR trend with time is the health-screening nature of volunteering. The huge shortfall in SMRs for the first year of UKCTOCS, particularly causes other than cancer, are strong indicators that women suffering from poor health or chronic non-cancer illness tend not to volunteer [4]. Their health concerns naturally lie with their immediate real problems rather than a future potential issue. Some of these conditions may well predispose to earlier mortality. It is interesting that the younger age groups, specifically 50-54, had higher SMRs. This suggests that women in the younger groups may more closely represent their national counterparts. We were unable to find in the literature reports where differences in (age-adjusted) SMRs were explored by age, but if confirmed, one possible explanation is that there may be less prevalent morbidity that might hinder volunteering at these ages. Mental and behavioural deaths had the lowest SMRs and the need for informed consent could have been a contributory factor. For general health, the commonly reported u-shaped curve [14-16] relating BMI to mortality was seen, with the highest SMRs belonging to those underweight and obese (Table 1).

The HVE is often ascribed to the fact that educated women who are financially better off and have a healthier lifestyle are more likely to volunteer for a screening trial, where health awareness and the means to travel to the trial centre influence a women's decision. While difficult to substantiate directly, many of these factors are linked to indices of social deprivation and in UKCTOCS the availability of postcodes for all invited women made it possible to calculate deprivation (IMD) scores for all invitees from England. Figure 1 shows that the cohort of invited women was more deprived (higher IMD scores: mean = 24.7) than those who subsequently joined the trial (mean score = 19.6). The reported mean IMD score for England is 21.6 [17]. Our invited population was more deprived than the national average probably as a result of a higher proportion of urban centres. However, as has been shown, those who actually volunteered were less deprived. Of note, Bristol and Portsmouth, which were the least deprived (lowest mean IMD) among the 10 English centres, had the highest acceptance rates of invitations among all 13 centres [5]. This suggests that postal invitation alone will not persuade women from deprived backgrounds to participate.

Socioeconomic status is known to be linked to most causes of mortality, including cancers. Bristol, which had the 2^nd ^lowest mean IMD score had the lowest SMR, while Liverpool, with the highest mean IMD score, had the highest SMR (48.4%). Further support is provided by the trend of higher SMRs with increasing levels of deprivation across the 5 quintiles, from the least deprived (SMR = 30.4%) to the most deprived (SMR = 52.3%) (Table 5).

The most striking aspect on comparing cause-specific incidence and mortality rates was that the SIRs were higher than the SMRs and, for the leading cancers other than lung, pancreas, oesophagus and colorectal (just), the SIR confidence intervals crossed 100. In a recent analysis of US data, while late-stage diagnoses in all cancers (with resultant higher mortality) were associated with lower socioeconomic status, incidence of only certain specific cancers varied with socioeconomic status [18]. Pollock et al noted that while mortality increased with deprivation among patients suffering from lung, breast and colorectal cancers in the South Thames area, for incidence this was only observed in lung cancer [19]. Official national statistics for England and Wales show a mixture of positive (notably lung and cervix), negative (breast, leukaemia) and zero association (colorectal) between different cancer type incidences and deprivation [20]. The three cancers (lung, oesophagus and pancreas) with the largest shortfalls in SIRs in UKCTOCS have a strong link to smoking [21-23]. Individuals with lower socio-economic status are more likely to be current smokers, physically inactive and obese [24]. In all three of these cancers, there are reports of negative correlation between incidence and socioeconomic status [18-20,25,26]. Conversely, in breast cancer the SIR was 102% whilst the SMR was 43%, in keeping with previously reported associations between higher socioeconomic status and higher incidence of localised breast cancer but lower regional breast cancer mortality [25,27]. Women who volunteer for a screening trial are more likely to attend for breast screening and to be diagnosed with early stage disease. Overdiagnosis of breast cancer in the screened population could also contribute to higher incidence but lower mortality [28].

Despite the strong similarity of results for overall mortality between UKCTOCS and the PLCO trial there is less commonality when cause-specific results are compared. While pancreatic cancer has the highest SMR in both studies, large discrepancies exist for cancers such as uterus (52% UKCTOCS versus 22% PLCO), stomach (75% versus 41%) and oesophagus (76% versus 41%). Given the smaller numbers in these subgroups, some of these differences may be purely random. Most of these cancers are also associated with lower SIRs in the PLCO trial compared to UKCTOCS: oesophageal (72% UKCTOCS versus 38% PLCO), stomach cancer (85% versus 48%) and bladder (80% versus 52%). It needs to be noted that there are subtle differences in the PLCO entry criteria when compared to UKCTOCS, such as minimum age (55 versus 50 in UKCTOCS) and inclusion of women who had undergone bilateral oophorectomy.

The most recently published statistics for mortality (for 2005, published 2006 [6]) and incidence (for 2006, published 2008 [7]) produced by ONS were used to calculate EMRs for the period 2001-2009 so the data can be considered broadly representative. An additional issue is that the 'national' mortality rates were based on data from England and Wales only but was used to calculate EMRs for the 13 579 women from Northern Ireland. There were also approximations involved in the actual calculations, such as the age-group mortality rates representing the midpoint of that group and specific age-adjusted rates estimated by use of a best-fitting simple model. The EMRs were also assumed to be fixed values when calculating the confidence intervals. They are estimates, as they are based on national data that varies yearly through a random component, in addition to any real change. However comparison of ONS's 2004 and 2005 (logged) mortality rates showed a high level of linear correlation, with all Pearson correlations for the major cancer causes over 0.99, except those for uterus (*r *= 0.984) and non-Hodgkin's lymphoma (*r *= 0.981). This suggests any yearly changes in mortality rates (real shifts or random fluctuations) are small and treating them as fixed was not unreasonable.

## Conclusions

The lack of mortality or incidence events can severely harm a clinical trial's ability to demonstrate efficacy. Other ramifications of the HVE inevitably include concerns over external validity of a demonstrated screening benefit, though that would imply some level of interaction between screening and volunteer characteristics. It may be hard to perceive how social factors could influence screening success directly at the point of intervention, though certainly compliance with a screening programme can be dependent upon the level of social deprivation [29]. Either way, one may regard this as a realistic aspect of a national screening programme. In UKCTOCS, the HVE has necessitated revision of the trial design in 2008, with extension of screening in the study arm until 31^st ^Dec 2011 and follow up until 31^st ^Dec 2014 [30]. During planning of this trial in 1999, no published data was available to estimate the impact of the HVE. The various mortality rates presented here are based on over one million study years, and incidence rates on over half a million study years. They provide vital information for investigators on likely event rate shortfalls that might be expected in ongoing and future screening studies/RCTs of similar design.

## Competing interests

IJ has consultancy arrangements with Becton Dickinson, who have an interest in tumour markers and ovarian cancer. They have provided consulting fees, funds for research, and staff but not directly related to this study. SS has received research support from Fujirebio Diagnostics but not in relation to this trial.

## Authors' contributions

IJ, UM, MP, SS, LF, SC were involved in trial design and concept. UM, MB, AR, MH AG-M and SA were involved in acquisition of data. MB, MP and UM were involved in the statistical analysis. MB and UM were responsible for interpretation of data and drafted the manuscript. All authors critically revised the manuscript and approved the final version. UM is the guarantor.

## Funding

The trial was core funded by the Medical Research Council (grant no. G990102), Cancer Research UK (grant no. C1479/A2884), and the Department of Health with additional support from the Eve Appeal, Special Trustees of Bart's and the London, and Special Trustees of UCLH. A major portion of this work was done at UCLH/UCL within the "women's health theme" of the NIHR UCLH/UCL Comprehensive Biomedical Research Centre supported by the Department of Health. SS has received research support from NCI (grant numbers CA086381 and CA083639). The researchers are independent from the funders.

Ethical approval: The study was approved by the UK North West Multicentre Research Ethics Committees (North West MREC 00/8/34) with site specific approval from the local regional ethics committees and the Caldicott guardians (data controllers) of the primary care trusts.

## References

[B1] Church TR Ederer F Mandel JS Watt GD Geisser MS Estimating the duration of ongoing prevention trials Am J Epidemiol 1993 137 7 797 810 8484371 10.1093/oxfordjournals.aje.a116740

[B2] Pinsky PF Miller A Kramer BS Church T Reding D Prorok P Gelmann E Schoen RE Buys S Hayes RB Berg CD Evidence of a healthy volunteer effect in the prostate, lung, colorectal, and ovarian cancer screening trial Am J Epidemiol 2007 165 8 874 81 10.1093/aje/kwk075 17244633

[B3] Britton A McKee M Black N McPherson K Sanderson C Bain C Choosing between randomised and non-randomised studies: a systematic review Health Technol Assess 1998 2 13 i iv 1-124 9793791

[B4] Ederer F Church TR Mandel JS Sample sizes for prevention trials have been too small Am J Epidemiol 1993 137 7 787 96 8484370 10.1093/oxfordjournals.aje.a116739

[B5] Menon U Gentry-Maharaj A Ryan A Sharma A Burnell M Hallett R Lewis S Lopez A Godfrey K Oram D Herod J Williamson K Seif M Scott I Mould T Woolas R Murdoch J Dobbs S Amso N Leeson S Cruickshank D McGuire A Campbell S Fallowfield L Skates S Parmar M Jacobs I Recruitment to multicentre trials--lessons from UKCTOCS: descriptive study BMJ 2008 337 a2079 10.1136/bmj.a2079 19008269 PMC2583394

[B6] ONS Mortality Statistics: Cause 2005 Office for National Statistics 2006 http://www.statistics.gov.uk/downloads/theme_health/Dh2_32/DH2_No32_2005.pdf Series DH2(Number 32)

[B7] ONS Cancer Statistics: Registrations 2006 Office for National Statistics 2008 http://www.statistics.gov.uk/downloads/theme_health/MB1-37/MB1_37_2006.pdf Series MB1(Number 37)

[B8] Noble M McLennan D Wilkinson K English Indices of Deprivation 2007 London: Communities and Local Government Publications 2008 http://www.communities.gov.uk/documents/communities/pdf/733520.pdf

[B9] Statistics for Wales Welsh Index of Multiple Deprivation (WIMD). Summary Report Welsh Assembly Government 2008 http://wales.gov.uk/docs/statistics/2010/100712wimd08summaryen.pdf

[B10] Brown AE Raynor P Benton D Lee MD Indices of Multiple Deprivation predict breastfeeding duration in England and Wales Eur J Public Health 2010 20 231 5 10.1093/eurpub/ckp114 19667052

[B11] Prorok PC Andriole GL Bresalier RS Buys SS Chia D Crawford ED Fogel R Gelmann EP Gilbert F Hasson MA Hayes RB Johnson CC Mandel JS Oberman A O'Brien B Oken MM Rafla S Reding D Rutt W Weissfeld JL Yokochi L Gohagan JK Prostate, Lung, Colorectal and Ovarian Cancer Screening Trial Project Team Design of the Prostate, Lung, Colorectal and Ovarian (PLCO) Cancer Screening Trial Control Clin Trials 2000 21 6 Suppl 273S 309S 10.1016/S0197-2456(00)00098-2 11189684

[B12] Rivera CM Grossardt BR Rhodes DJ Rocca WA Increased mortality for neurological and mental diseases following early bilateral oophorectomy Neuroepidemiology 2009 33 1 32 40 10.1159/000211951 19365140 PMC2697609

[B13] Rivera CM Grossardt BR Rhodes DJ Brown RD Jr Roger VL Melton LJ Rocca WA Increased cardiovascular mortality after early bilateral oophorectomy Menopause 2009 16 1 15 23 10.1097/gme.0b013e31818888f7 19034050 PMC2755630

[B14] Lewis CE McTigue KM Burke LE Poirier P Eckel RH Howard BV Allison DB Kumanyika S Pi-Sunyer FX Mortality, health outcomes, and body mass index in the overweight range: a science advisory from the American Heart Association Circulation 2009 119 25 3263 71 10.1161/CIRCULATIONAHA.109.192574 19506107

[B15] Hanson RL McCance DR Jacobsson LT Narayan KM Nelson RG Pettitt DJ Bennett PH Knowler WC The U-shaped association between body mass index and mortality: relationship with weight gain in a Native American population J Clin Epidemiol 1995 48 7 903 16 10.1016/0895-4356(94)00217-E 7782799

[B16] Whitlock G Lewington S Sherliker P Clarke R Emberson J Halsey J Qizilbash N Collins R Peto R Body-mass index and cause-specific mortality in 900 000 adults: collaborative analyses of 57 prospective studies Lancet 2009 373 9669 1083 96 10.1016/S0140-6736(09)60318-4 19299006 PMC2662372

[B17] ONS ID 2007 Average IMD Score (Population weighted average of the combined index of multiple deprivation (IMD) scores for the SOAs in a district) Office for National Statistics 2007 http://data.gov.uk/dataset/id_2007_average_imd_score

[B18] Clegg LX Reichman ME Miller BA Hankey BF Singh GK Lin YD Goodman MT Lynch CF Schwartz SM Chen VW Bernstein L Gomez SL Graff JJ Lin CC Johnson NJ Edwards BK Impact of socioeconomic status on cancer incidence and stage at diagnosis: selected findings from the surveillance, epidemiology, and end results: National Longitudinal Mortality Study Cancer Causes Control 2009 20 4 417 35 10.1007/s10552-008-9256-0 19002764 PMC2711979

[B19] Pollock AM Vickers N Breast, lung and colorectal cancer incidence and survival in South Thames Region, 1987-1992: the effect of social deprivation J Public Health Med 1997 19 3 288 94 9347452 10.1093/oxfordjournals.pubmed.a024632

[B20] Quinn M Babb P Brock A Kirby E Jones J Cancer trends in England and Wales, 1950-1999 Office for National Statistics, Studies on Medical and Population Subjects 2001 66

[B21] Loeb LA Ernster VL Warner KE Abbotts J Laszlo J Smoking and lung cancer: an overview Cancer Res 1984 44 12 Pt 1 5940 58 6388830

[B22] Michaud DS Epidemiology of pancreatic cancer Minerva Chir 2004 59 2 99 111 15238885

[B23] Blot WJ McLaughlin JK The changing epidemiology of esophageal cancer Semin Oncol 1999 26 5 Suppl 15 2 8 10566604

[B24] Schoenborn CA Adams PF Barnes PM Vickerie JL Schiller JS Health behaviors of adults: United States, 1999-2001 Vital Health Stat 2004 10 219 1 79 15791896

[B25] Faggiano F Partanen T Kogevinas M Boffetta P Socioeconomic differences in cancer incidence and mortality IARC Sci Publ 1997 138 65 176 9353664

[B26] Wood HE Gupta S Kang JY Quinn MJ Maxwell JD Mudan S Majeed A Pancreatic cancer in England and Wales 1975-2000: patterns and trends in incidence, survival and mortality Aliment Pharmacol Ther 2006 23 8 1205 14 10.1111/j.1365-2036.2006.02860.x 16611282

[B27] Yabroff KR Gordis L Does stage at diagnosis influence the observed relationship between socioeconomic status and breast cancer incidence, case-fatality, and mortality? Soc Sci Med 2003 57 12 2265 79 10.1016/S0277-9536(03)00100-X 14572836

[B28] Gotzsche PC Hartling OJ Nielsen M Brodersen J Jorgensen KJ Breast screening: the facts--or maybe not BMJ 2009 338 b86 10.1136/bmj.b86 19174442

[B29] Zackrisson S Lindstrom M Moghaddassi M Andersson I Janzon L Social predictors of non-attendance in an urban mammographic screening programme: a multilevel analysis Scand J Public Health 2007 35 5 548 54 10.1080/14034940701291716 17852976

[B30] Menon U Jacobs I Protocol for the United Kingdom Collaborative Trial of Ovarian Cancer Screening 2008 http://www.instituteforwomenshealth.ucl.ac.uk/academic_research/gynaecologicalcancer/gcrc/ukctocs/files/Protocol-v4.pdf

